# Influence of dilution on arterial-phase artifacts and signal intensity on gadoxetic acid–enhanced liver MRI

**DOI:** 10.1007/s00330-022-08984-0

**Published:** 2022-07-27

**Authors:** Sarah Poetter-Lang, Gregor O. Dovjak, Alina Messner, Raphael Ambros, Stephan H. Polanec, Pascal A. T. Baltzer, Antonia Kristic, Alexander Herold, Jacqueline C. Hodge, Michael Weber, Nina Bastati, Ahmed Ba-Ssalamah

**Affiliations:** Department of Biomedical Imaging and Image-Guided Therapy, General Hospital of Vienna (AKH), Medical University of Vienna, Waehringer Guertel 18-20, 1090 Vienna, Austria

**Keywords:** Liver, Magnetic resonance imaging, Gadolinium ethoxybenzyl DTPA, Artifacts, Signal intensity

## Abstract

**Objectives:**

To investigate the effect of saline-diluted gadoxetic acid, done for arterial-phase (AP) artifact reduction, on signal intensity (SI), and hence focal lesion conspicuity on MR imaging.

**Methods:**

We retrospectively examined 112 patients who each had at least two serial gadoxetic acid–enhanced liver MRIs performed at 1 ml/s, first with non-diluted (ND), then with 1:1 saline-diluted (D) contrast. Two blinded readers independently analyzed the artifacts and graded dynamic images using a 5-point scale. The absolute SI of liver parenchyma, focal liver lesions (if present), aorta, and portal vein at the level of the celiac trunk and the SI of the paraspinal muscle were measured in all phases. The signal-to-norm (SI_Norm_) of the vascular structures, hepatic parenchyma and focal lesions, and the contrast-to-norm (*C*_Norm_) of focal liver lesions were calculated.

**Results:**

AP artifacts were significantly reduced with dilution. Mean absolute contrast-enhanced liver SI was significantly higher on the D exams compared to the ND exams. Likewise, SI_Norm_ of liver parenchyma was significantly higher in all contrast-enhanced phases except transitional phase on the D exams. SI_Norm_ values in the AP for the aorta and in the PVP for portal vein were significantly higher on the diluted exams. The *C*_Norm_ was not significantly different between ND and D exams for lesions in any imaging phase. The interclass correlation coefficient was excellent (0.89).

**Conclusion:**

Gadoxetic acid dilution injected at 1ml/s produces images with significantly fewer AP artifacts but no significant loss in SI_Norm_ or *C*_Norm_ compared to standard non-diluted images.

**Key Points:**

*• Diluted gadoxetic acid at slow injection (1 ml/s) yielded images with higher SI*_*Norm*_ *of the liver parenchyma and preserved* *C*_*Norm*_ *for focal liver lesions.*

*• Gadoxetic acid–enhanced MRI injected at 1 ml/s is associated with arterial-phase (AP) artifacts in 31% of exams, which may* *degrade image quality and limits focal liver lesion detection.*

*• Saline dilution of gadoxetic acid 1:1 combined with a slow injection rate of 1 ml/s significantly reduced AP artifacts from 31 to 9% and non-diagnostic AP artifacts from 16 to 1%.*

## Introduction

Gadoxetic acid (gadolinium ethoxybenzyl DTPA)–enhanced magnetic resonance imaging (MRI) is increasingly used for detection and characterization of focal liver lesions [1, 2]. However, one of gadoxetic acid’s major drawbacks is arterial-phase (AP) artifacts which may degrade image quality, limiting diagnosis of focal liver lesions [3–5].

Two types of AP artifacts have been reported during gadoxetic acid–enhanced MR imaging. The first is transient severe motion (TSM) due to acute fleeting elevation of peak gadoxetic acid blood plasma concentration and is associated with acute transient dyspnea [6, 7]. The second, truncation artifacts, may be related to the relatively rapid disappearance of contrast from the imaging field. Both small volumes (≤ 10 ml) and recommended injection rates (e.g., 2 ml/s) yield a compact contrast bolus and rapid changes in intravascular gadoxetic acid concentration during k-space data acquisition [8, 9].

To overcome these artifacts, several strategies have been developed [9–15]. Two of the most promising techniques are saline dilution of gadoxetic acid at 1:1 and slow injection rate of 1 ml/s. Both are off-label use as the suggested dose is undiluted 0.1mmol/kg at 2 ml/s. Both stretch the bolus length, and lower the peak plasma gadoxetic acid concentration during the arterial phase [7, 14, 16]. In turn, lower peak plasma concentrations may fall below the threshold that triggers central chemoreceptors, thus preventing hyperventilation and minimizing TSM, as suggested by Polanec et al [7]. Furthermore, the prolonged arterial phase improved uniformity of contrast bolus during the acquisition allowing more homogeneous filling of central k-space. Therefore, diluted gadoxetic acid injected at 1 ml/s, following a test bolus tracking technique, has been shown to reduce TSM artifacts significantly [7]. However, we wondered if decreasing the peak contrast concentration through dilution and slower injection rate would also cause SI loss yielding suboptimal images with low signal or contrast values, impairing focal liver lesion detection, the main diagnostic task.

Thus, our primary aim was to investigate the effect of dilution and slow injection rate of 1 ml/s on quantitative differences in absolute signal intensity, signal-to-norm, and contrast-to-norm on MR images in the arterial, portal venous, transitional, and 20-min hepatobiliary (HB) phases. Secondly, we also evaluated the non-diluted (ND) and saline-diluted (D) images post-intravenous gadoxetic acid for AP artifacts.

## Materials and methods

### Patients

**Fig. 1:**
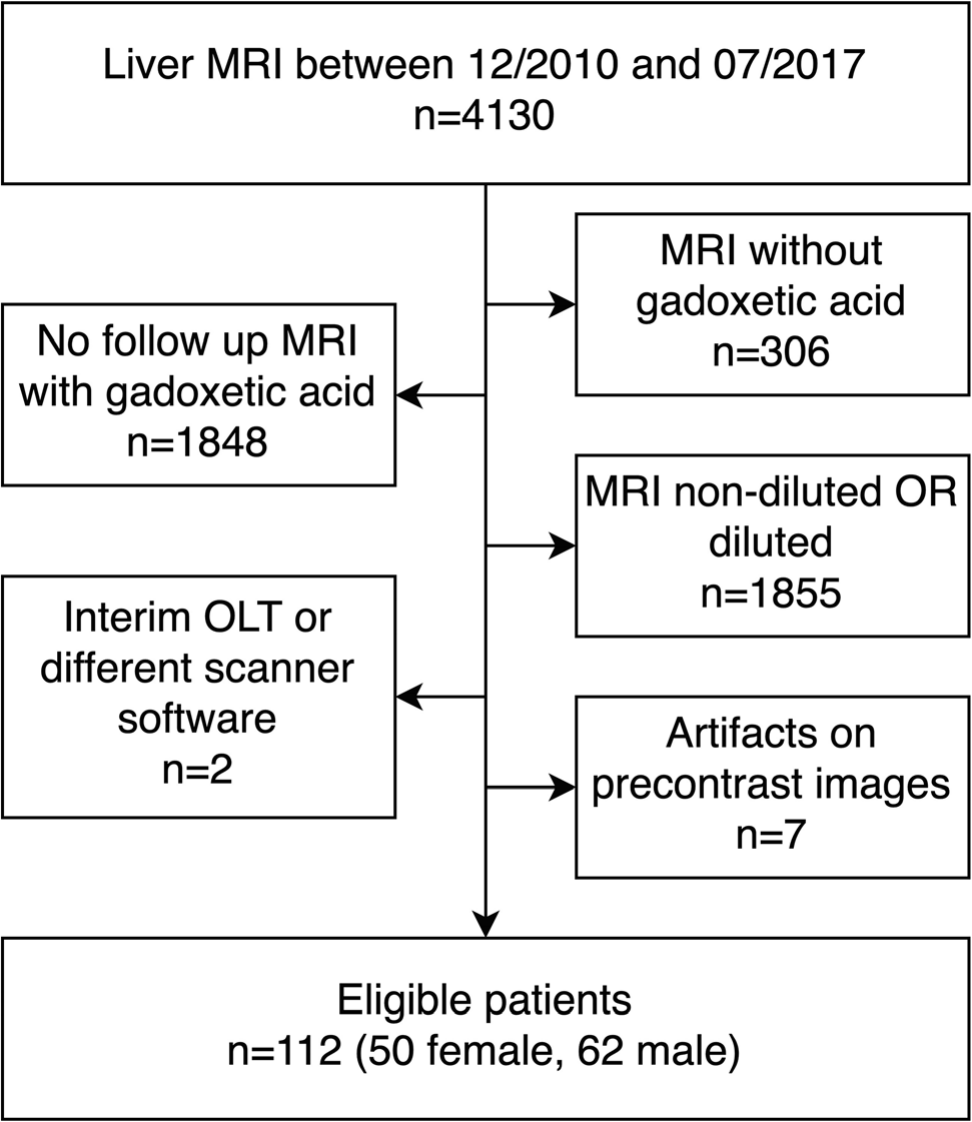
Flowchart of the exclusion criteria yielding eligible patients. MRI = magnetic resonance imaging

For this single-center retrospective study, designed according to STROBE (Strengthening the Reporting of Observational Studies in Epidemiology), our institutional ethics review board approved the data collection and analysis and waived the requirement for informed consent. A search in our picture archiving and communication system (PACS) database identified 4130 consecutive liver MRIs performed for known or suspected liver or pancreaticobiliary diseases between December 2010 and July 2017 (Fig. 1). One hundred twenty-one (*n* = 121) patients had undergone at least one non-diluted (ND) between December 2010 and 2014 and one saline-diluted (D) between January 2015 and July 2017 power-injected gadoxetic acid–enhanced MRI, at 1 ml/s with identical exam parameters. Patients were excluded if they had undergone liver transplantation between the two MRIs (*n* = 1). We further excluded one patient that had been studied before the software update. Seven additional patients were excluded because they had artifacts in all sequences, including non-contrast images. If patients had several serial MRI exams, we took the most recent non-diluted MRI and compared with the first diluted MRI exam, i.e., we took the two MRIs that were closest in proximity to one another.

### MR examination protocol

All patients underwent a 3 Tesla liver MRI (Magnetom, Trio Tim, Siemens Medical Systems) using a combined six-element, phased-array abdominal coil and a fixed spine coil to improve SNR. Unenhanced and contrast-enhanced imaging was performed using fat-suppressed T1-weighted three-dimensional gradient-echo volumetric interpolated breath-hold examination (VIBE) sequence. VIBE imaging parameters for both the diluted and non-diluted patients were as follows: TR 2.67 ms, TE 0.97 ms, phase direction AP, and flip angle 13°. Depending upon the patient size, slice thickness (1.7 to 2 mm), matrix size (288 × 168 to 512 × 384), FOV (380 to 430 mm), and number of slices (90 to 120) varied. Parallel imaging with an acceleration factor of two was used in both groups. Matrix and slice thickness given are for the reconstruction using zero interpolation. The prescan normalization filter provided by the vendor was activated in all sequences to homogenize the SI.

An intravenous (IV) bolus of gadoxetic acid was administered at a dosage of 0.025 mmol/kg body weight (0.1 ml/kg body weight) through a 20- to 22-gauge antebrachial venous catheter. Dynamic images were obtained at t-peak plus 5 s (arterial), 70 s (portal venous), 300 s (transitional phase), and 20 min, i.e., hepatobiliary phase (HBP) with the identical parameters used for unenhanced sequence; t-peak determination is described below. Our k-space ordering was sequential. The acquisition time per sequence ranged from 14 to 20 s, depending on patient size. The exam protocol also included axial in- and opposed-phase T1-weighted images, T2-weighted HASTE, and diffusion-weighted images. There was one software upgrade during the 7-year study period, which affected a single patient that was subsequently excluded from the cohort. Scanner and imaging parameters for both groups (D and ND) were unaltered.

### Contrast media injection techniques

All test doses were 0.5 ml of gadoxetic acid injected at 1 ml/s; the bolus was flushed with 25 ml saline via a power injector (Spectris Solaris, MR; Medrad Europe) at the same flow rate. During the test injection, T1-weighted GRE 2D axial images were obtained at one frame/s for 60 s, thus capturing multiple arterial, mixed, and portal venous phase images during gadoxetic acid’s passage through the aorta. Based upon these preliminary images, the post-injection time in which the SI of the aorta at the level of the celiac artery appeared highest was selected as the time to peak aortic enhancement (t-peak) for that patient. Next, 0.025 mmol/kg body weight of gadoxetic acid was administered as an IV bolus at 1 ml/s, ND (from December 2010 to December 2014). Aiming to reduce artifacts, we started administering an IV bolus of 1:1 saline-diluted (D) gadoxetic acid in January 2015, administered as an IV bolus at the same rate of 1 ml/s. The duration of contrast injection depended upon the contrast volume. For example, a non-diluted 10-ml volume required 10 s, whereas when diluted, the 20 ml volume took 20 s. Then, dynamic imaging was performed as described above, with arterial-phase imaging initiated at the individual patient’s t-peak, as determined by the test injection plus 5 s. We used an automated voice recording to give patients breathing instructions.

### Image analysis

Dynamic T1-weighted sequences (unenhanced, arterial, portal venous, transitional, and 20-min HB phases) were reviewed by two readers (G.D. and S.P.) independently, each with at least 5 years of experience in liver MRI, and the presence of artifacts was graded on a 5-point scale, as described before [17]: (1) no artifacts, (2) minimal artifacts, without diagnostic effect, (3) moderate artifacts, with minimal diagnostic effect, (4) marked artifacts, with significant diagnostic effect, and (5) severe artifacts, non-diagnostic. Seven patients with artifacts in all sequences were excluded on the assumption that these artifacts were not solely injection-related artifacts. AP artifacts were defined as a score ≥ 4 during the arterial phase [4]. To reach the final artifact score, the scores of readers 1 and 2 were averaged and rounded up.

**Fig. 2:**
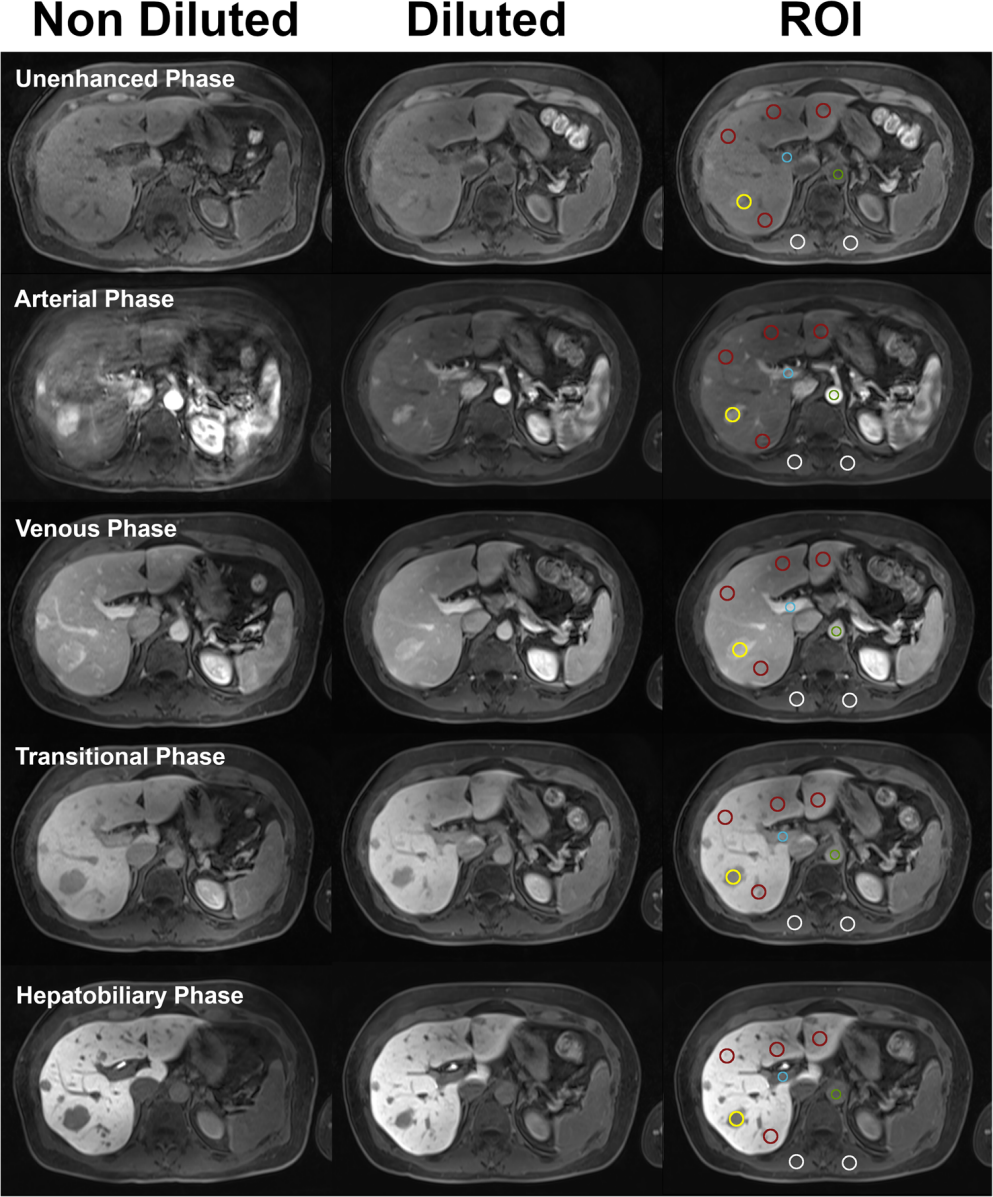
Three columns of axial liver MR images showing non-diluted and diluted injections in the same patient. The last column shows the regions of interest (ROI) as measured in the five specified locations: aorta (green), portal vein (blue), liver parenchyma (red), focal liver lesion (yellow), and paraspinal muscle (white) in all phases

Next, quantitative image analysis was performed by the same two readers (G.D. and S.P.) independently and blinded to the contrast injection method. Images were randomly reviewed on a dedicated PACS workstation. Quantitative signal intensity (SI) measurements were acquired by drawing regions of interest (ROI) at the level of the celiac trunk. SI was recorded, placing the ROI as follows [18]: for the aorta, main portal vein ≥ 0.5 cm^2^; for the right anterior, right posterior, left medial, and left lateral liver segments ≥ 2 cm^2^; and for paraspinal muscle 1–2 cm^2^. Finally, for patients with visible liver lesions ≥ 1 cm^2^, deemed comparable between the two exams, a ROIs was drawn to encompass the largest lesion in its entirety (Fig. 2). For patients with comparable lesions of more than one etiology, the largest was selected for SI measurements. No ROIs were placed in hepatic parenchyma containing large vessels. The same ROIs were chosen and copied to identical positions in all imaging phases (unenhanced, arterial, portal venous, transitional, and HB) for each patient. To normalize the obtained SI values, we chose to use the SI of the paraspinal muscle as a reference to calculate the signal-to-norm ratio (SI_Norm_) [19]. The SI_Norm_ was calculated for each ROI using the equation:${SI}_{Norm}(i)=\frac{SI_i}{SI_{Muscle}}$, where *i* is the tissue being measured. Lastly, the contrast-to-norm ratio (*C*_Norm_), referring to focal liver lesion conspicuity, was calculated using the equation: ${C}_{Norm}= abs\left(\frac{SI_{liver}-{SI}_{lesion}}{SI_{muscle}}\right)= abs\left({SI}_{Norm}(liver)-{SI}_{Norm}(lesion)\right)$, on unenhanced, arterial, portal venous, transitional, and HB phases, respectively. The absolute difference was chosen to obtain a value that represents the visible difference between liver parenchyma and focal lesion, whether hypointense or hyperintense.

### Statistical analysis

For this study, our null hypothesis was that diluted exams, performed for AP artifact reduction, do not differ in the SI and SI_Norm_ of the vascular structures and liver parenchyma as well as *C*_Norm_ of focal liver lesions. Our alternative hypothesis was that dilution would lead to SI, SI_Norm_, and *C*_Norm_ decrease or increase in comparison to ND scans. All statistical analyses were performed with commercially available software. For data collection and management, an Excel (Microsoft Corporation) spreadsheet was used. The statistical analysis was performed with SPSS Statistics version 26 (IBM).

Patient characteristics that were continuous variables (e.g., patient age, BMI) were summarized with means and standard deviation. Categorical variables (e.g., patient gender, presence of ascites, and/or cirrhosis) were summarized with counts and percentages. The inter-rater variability was assessed by two-way mixed intraclass correlation coefficient (ICC) with an absolute agreement for quantitative SI differences and using Cohen’s kappa (*κ*) coefficient for qualitative artifacts interpretation. We performed a paired *t*-test for comparison of the means of ND and D exams to assess the effect of dilution on SI and SI_Norm_. To compare *C*_Norm_ of focal liver lesions, only subgroups with *n* ≥ 10 were chosen. To compare signal intensities between the different artifact grade groups, Welch’s corrected one-way ANOVAs were used for the ND and D groups separately, grouping grades 4 and 5 because of their small number. As the design of this study was not confirmatory, the beta error (sensitivity to find statistical significance) is considered worse than the alpha error (specificity of statistical significances). Therefore, no formal correction for multiple testing was used. Related samples Wilcoxon’s signed-rank test was used to compare artifact grades between D and ND. Fisher’s exact test was used to compare the sizes of groups with AP artifacts with those without AP artifacts between ND and D exams. Values of *p* < 0.05 were considered statistically significant.

## Results

The demographics of the 112 study patients (50 female and 62 male) are listed in Table 1. The mean time interval between the two MRI examinations was 746 days (range 199–1828 days). All MRIs were performed with the same scanner, software, and examination protocol. The sole exception was contrast media application, at 1 ml/s, which was either diluted or non-diluted. The mean patient age at the first MRI exam was 54.8 years (21–85 years) and the mean BMI was 25.3 kg/m^2^ (17.7–35.5 kg/m^2^). At follow-up exam, the mean age was 56.9 (23–85 years) and mean BMI 25.2 kg/m^2^ (17.5–39.1 kg/m^2^, *p* = 0.922). For the disease severity in cirrhotic patients, there was no significant interval change in the Child-Pugh score (5.85 versus 6.04, *p* = 0.612) and MELD score (6.05 versus 7.35, *p* = 0.218). Regarding renal function, there was no significant change in the interim for GFR (97.67 vs 94.34, *p* = 0.37). No cardiac events were noted in the medical record for any patient during the study period.

**Table 1:** Demographic patient data. One HCC (LIRADS 3-5 lesion) and one metastasis developed in between scans in patients where no lesion was reported priorly. One adenoma developed in a treated lesion. One patient with a treated lesion developed recurrent HCC. Seven HCC lesions and eight metastases developed into treated lesions

Patient characteristic	Non-diluted (ND)	Diluted (D)
Number of included patients	112	112
Number of females	50 (44.6%)	50 (44.6%)
Number of males	62 (55.4%)	62 (55.4%)
Mean age	54.8 (14.2)	56.9 (14.1)
Mean body mass index (kg/m^2^)	25.3 (4.2)	25.2 (4.4)
Liver cirrhosis	35 (31.3%)	35 (31.3%)
Moderate or severe ascites	12 (10.7%)	11 (9.8%)
Patients with a liver lesion	68 (60.7 %)	70 (62.5%)
Adenoma	7 (6.3%)	6 (5.4%)
FNH	12 (10.7%)	12 (10.7%)
Cirrhotic nodules (LIRADS 3-5)	20 (17.9%)	15 (13.4%)
Metastases	23 (20.5%)	16 (14.3%)
Hemangioma	3 (2.7%)	3 (2.7%)
Treated lesions	3 (2.7%)	18 (16.1%)

### Arterial-phase artifacts (AP artifacts)

Significantly more AP artifacts were observed in the non-diluted exams (35 patients, 31%) compared to the diluted exams (ten, 9%). An artifact severity score of 5 (non-diagnostic) was assigned to 18 exams (16%). All but one (1%) of 18 (16%) exams with an artifact severity score of 5 (non-diagnostic) were in the non-diluted group. The severity of the artifacts scored significantly higher in the ND compared to the D protocol (*p* < 0.001).

### Signal intensity measurements

On the unenhanced liver MRIs, there were no statistically significant differences in SI and SI_Norm_ between the ND and D exams in the aorta, liver, PV, or lesion (Tables 2 and 3). The mean absolute liver parenchyma SI was significantly higher for the D exams as compared to the ND exams, on arterial, portal venous, transitional, and HB phases (*p* = 0.012, *p* = 0.002, *p* = 0.019, and *p* < 0.001, respectively) (Table 2).

**Table 2:** Detailed results of SI, i.e., the mean SI values of the liver in manually placed ROI calculated from the average reported by readers 1 and 2. Significant differences, *p* < 0.05, are highlighted in bold. The *p* value of comparison by paired *t*-test is given. *PV* portal vein. *SD* standard deviation. *SI* signal intensity

		Non-diluted (ND)		Diluted (D)		
		Mean SI	SD	Mean SI	SD	*p* value
Unenhanced	Liver	256.0	46.2	264.0	47.3	0.069
	Aorta	178.0	46.2	176.2	41.4	0.679
	PV	177.4	34.3	177.1	38.9	0.939
Arterial phase	Liver	308.6	69.9	327.8	73.5	**0.012**
	Aorta	1064.8	332.7	1155.6	351.8	**0.006**
	PV	576.2	241.5	576.3	249.4	0.997
Portal venous phase	Liver	410.2	89.7	437.2	101.6	**0.002**
	Aorta	712.1	200.9	738.1	206.7	0.073
	PV	773.3	204.8	821.3	234.9	**0.007**
Transitional phase	Liver	419.4	100.4	442.4	114.4	**0.019**
	Aorta	492.8	130.9	505.6	143.0	0.193
	PV	500.3	121.0	519.6	130.6	0.094
Hepatobiliary phase	Liver	452.4	105.2	499.1	129.6	**< 0.001**
	Aorta	367.2	99.7	371.4	103.2	0.605
	PV	366.5	88.1	371.6	93.2	0.556

**Table 3:** Detailed results of signal-to-norm (SI_Norm_), i.e., the mean SI values of the liver divided by the SI of the paraspinal muscle in manually placed ROI calculated from the average reported by readers 1 and 2. Significant differences, *p* < 0.05, are highlighted in bold. The *p* value by paired *t*-test is given. *PV* portal vein. *SD* standard deviation. The SI_Norm_ was calculated for each ROI using the equation: SI_Norm_ = SI_liver_/SI_muscle_

		Non-diluted (ND)		Diluted (D)		
		Mean SI_Norm_	SD	Mean SI_Norm_	SD	*p* value
Unenhanced	Liver	1.24	0.24	1.25	0.22	0.750
	Aorta	0.86	0.20	0.83	0.16	0.073
	PV	0.86	0.17	0.83	0.18	0.191
Arterial phase	Liver	1.28	0.26	1.38	0.30	**0.002**
	Aorta	4.44	1.40	4.85	1.40	**0.005**
	PV	2.39	0.95	2.42	1.02	0.794
Portal venous phase	Liver	1.63	0.30	1.71	0.37	**0.010**
	Aorta	2.82	0.70	2.88	0.71	0.263
	PV	3.08	0.76	3.23	0.91	**0.035**
Transitional phase	Liver	1.63	0.32	1.66	0.40	0.364
	Aorta	1.01	0.20	1.00	0.19	0.569
	PV	1.95	0.44	1.95	0.45	0.888
Hepatobiliary phase	Liver	1.90	0.40	2.04	0.47	**< 0.001**
	Aorta	1.54	0.39	1.52	0.40	0.599
	PV	1.54	0.37	1.53	0.36	0.564

**Fig. 3:**
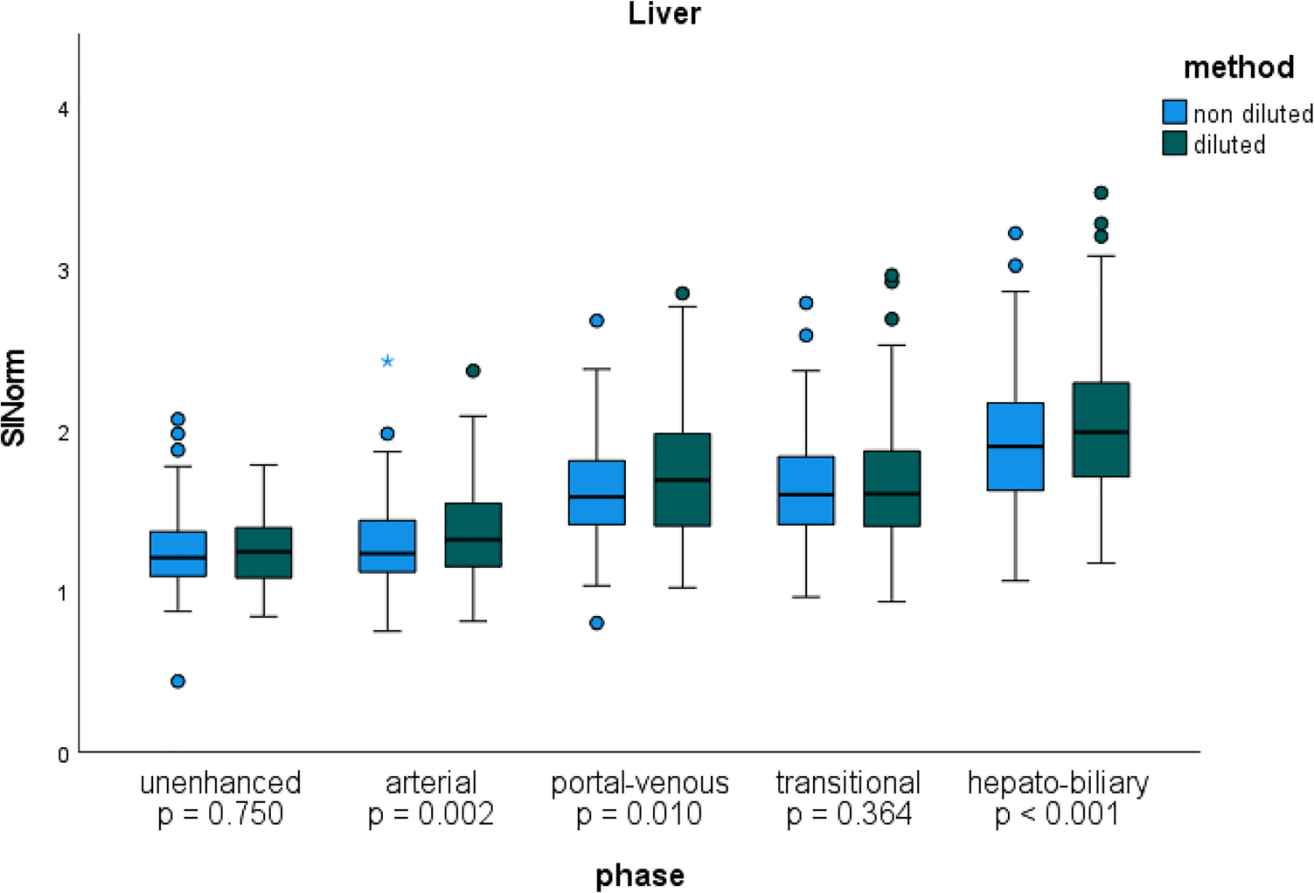
SI_Norm_. Boxplot graphic of liver parenchyma signal intensity normalized to muscle SI (SI_Norm_) in all phases. The blue boxes represent exams performed with non-diluted contrast media, the green boxes diluted contrast media. The SI_Norm_ was found to be significantly higher with the diluted injection protocol in the arterial, portal venous, and hepatobiliary phases

Furthermore, mean liver SI_Norm_ was significantly higher on the D exams compared to the ND exams in the arterial, portal venous, and hepatobiliary phases (*p* = 0.002, *p* = 0.010, and *p* < 0.001), respectively (Table 3 and Fig. 3). The greatest difference was observed in the HBP. Furthermore, the mean SI_Norm_ of the aorta in the AP and of the portal vein in the portal venous phase was significantly higher in the D exams (*p* = 0.005 and *p* = 0.035), respectively (Table 3).

**Fig. 4:**
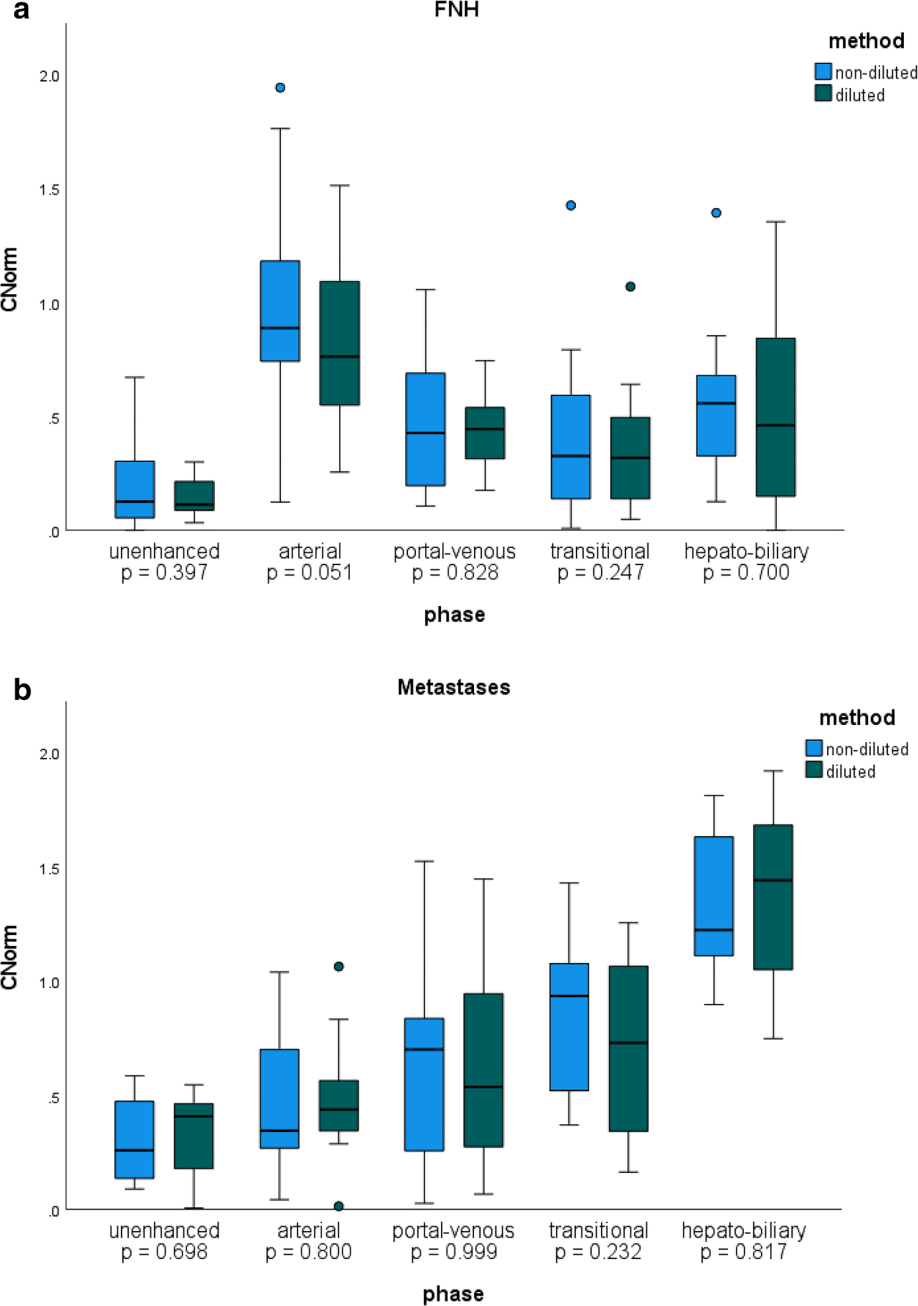
**a** C_Norm_ FNH. Boxplot graphic of the absolute difference between SI_Norm_ of FNHs and SI_Norm_ of the liver, called contrast-to-norm (C_Norm_) in all phases. The blue boxes represent exams performed with non-diluted contrast media, the green boxes diluted contrast media. There were no significant differences between the non-diluted and the diluted injection protocol in all phases. **b** C_Norm_ metastases. Boxplot graphics of the absolute difference between SI_Norm_ of metastases and SI_Norm_ of the liver, called contrast-to-norm (C_Norm_) in all phases. The blue boxes represent exams performed with non-diluted contrast media, the green boxes diluted contrast media. There were no significant differences between the non-diluted and the diluted injection protocol in all phases

There were 68 (60.7%) focal liver lesions on the ND exams of the 112 patients (Table 1), 48.5% of which were excluded due to interim change between ND and D exams. The etiology of the 35 comparable (51.5%) lesions included 12 FNH (34.3%), 11 metastases (31.4%), 5 cirrhotic nodules (cirrhotic nodules, i.e., LIRADS 3–5 lesions) (14.3%), 4 adenomas (11.4%), and 3 hemangiomas (8.6%). There was no significant difference in the *C*_Norm_ of all lesions combined for D versus ND exams (all phases *p* > 0.05). Evaluation of lesion subtype, when *n* ≥ 10 (i.e., FNH and metastases), also revealed no significant difference in *C*_Norm_ between the ND and D exams (all phases *p* > 0.05) (Table 4, Fig. 4).

**Table 4:** Detailed results of different lesion types using the mean and standard deviation of the *C*_Norm_ values, calculated from the average manually placed ROI by readers 1 and 2 in liver, lesion, and paraspinal muscle. *PV* portal vein. *SD* standard deviation. ${C}_{Norm}=\frac{abs\left({SI}_{liver}-{SI}_{lesion}\right)}{SI_{muscle}}= abs\left(\frac{SI_{liver}}{SI_{muscle}}-\frac{SI_{lesion}}{SI_{muscle}}\right)$. Paired *t*-tests were calculated for group sizes of *n* > 10 and *p* values are given, where *p* < 0.05 is significant

		Non-diluted (ND)		Diluted (D)		
		Mean *C*_Norm_	SD	Mean *C*_Norm_	SD	*p* value
Unenhanced	All lesions	0.25	0.19	0.26	0.19	0.850
	FNH	0.19	0.19	0.14	0.09	0.397
	Metastases	0.31	0.19	0.33	0.19	0.698
Arterial phase	All lesions	0.69	0.50	0.67	0.38	0.691
	FNH	1.01	0.49	0.83	0.42	0.051
	Metastases	0.46	0.33	0.49	0.28	0.800
Portal venous phase	All lesions	0.53	0.39	0.59	0.40	0.357
	FNH	0.48	0.32	0.45	0.17	0.828
	Metastases	0.63	0.49	0.63	0.43	0.999
Transitional phase	All lesions	0.60	0.39	0.59	0.42	0.791
	FNH	0.42	0.39	0.36	0.29	0.247
	Metastases	0.85	0.34	0.71	0.40	0.232
Hepatobiliary phase	All lesions	0.94	0.56	1.01	0.62	0.207
	FNH	0.56	0.34	0.52	0.44	0.700
	Metastases	1.35	0.34	1.38	0.39	0.817

### Influence of the artifacts on signal intensities

Arterial-phase artifacts did not have a significant influence on the signal intensity of liver parenchyma on the ND (*p* = 0.627) and on D (*p* = 0.644) exams. The vast majority of the arterial-phase artifacts in our cohort were TSM. No truncation artifacts affected diagnostic efficacy. To assess the effect of artifacts on lesion visibility, aside from *C*_Norm_, we looked at the visibility of lesions less than 1 cm. Because patients with malignant lesions underwent treatment between the MRIs, i.e., resection or ablation, we only compared benign lesions, e.g., FNH, hemangioma, and adenomas, < 1 cm. We found 8 such lesions. We were able to detect 5 more lesions and to see 3 lesions better on the diluted vs non-diluted scans. We attribute these findings to TSM artifact reduction as truncation artifacts did not influence the visibility of these lesions.

### Inter-reader agreement

The overall inter-reader agreement measured with the ICC for quantitative SI measurements in the arterial, portal venous, transitional, and HB phases was excellent (0.89). Also, the Cohen’s kappa (*κ*) coefficient for the visual evaluation of AP artifacts was also similarly high (0.831, *p* < 0.001).

## Discussion

Arterial-phase transient severe motion artifacts on gadoxetic acid–enhanced MRI can be mitigated by combined 1:1 saline dilution and slow injection rate of 1 ml/s while preserving or even increasing signal intensity. Furthermore, lesion contrast, indicated by *C*_Norm_, was not shown to be inferior on the diluted as compared to the non-diluted exams.

Although both dilution (1:1) and slow injection rate (at 1 ml/s) are off-label use of gadoxetic acid, this combined technique quadruples the time of the arterial phase, allowing k-space more time to achieve homogeneity [20]. Thus, the signal intensity could be higher in comparison to the recommended injection of non-diluted contrast at 2 ml/s.

The reduction of AP artifacts using either dilution or slow injection is already well-recognized. Our findings corroborate those of previous researchers [7, 14–16, 21, 22].

We too postulated that by doubling gadoxetic acid’s mean transit time relative to the short arterial-phase acquisition time, a more favorable bolus configuration was achieved, reducing truncation artifacts which may occur when small volumes, i.e., ≤ 10 ml, of contrast are injected [7, 14, 18, 23–25]**.** Furthermore, by using parallel imaging, we were able to shorten the acquisition time, making it more similar to the bolus transit time, thus improving k-space homogeneity [26]. AP artifacts were not shown to have a significant influence on SI or SI_Norm_ of the liver. Therefore, we attribute the higher SI and SI_Norm_ in the diluted exams to contrast dilution and slower injection rate.

Without dilution, AP artifacts have been observed in 10.7–39% of patients [3–6, 10–13]. Polanec et al [7] speculated that this transiently high gadoxetic acid concentration in serum may exceed the threshold, triggering central chemoreceptors, resulting in hyperventilation.

With combined dilution and slow injection, we likely kept peak plasma concentration below the tipping point, avoiding activation of central chemoreceptors that would cause breath-hold failure and possibly TSM artifacts.

We hypothesized that dilution and slow injection would further lower peak gadoxetic acid plasma concentration, which is already at a dose of 25% that of conventional gadolinium chelates reducing absolute SI, SI_Norm_, and *C*_Norm_, potentially decreasing focal liver lesion detection.

Surprisingly, dilution and slow injection did not cause the expected drop in SI in either absolute SI or SI_Norm_, and in fact increased aortic SI in the AP, portal vein SI in the PVP, and hepatic parenchymal SI in all phases. Furthermore, liver parenchymal SI_Norm_ increased on D versus ND exams in all but the transitional phase. However, once recirculation commenced, with each pass, the D exams maintained their lead on the ND exams which explains why the difference in SI and SI_Norm_ was largest in the HBP, indicating the widening gap between D and ND. Because the half-life of gadoxetic acid is circa 1 h, at this time frame, there is still about 50% of contrast still recirculating [27–29].

This increase in SI_Norm_, in the arterial, portal venous, and hepatobiliary phases on D compared to the ND groups, we attribute to the following factors: dilution, slow injection, and the high relaxivity of gadoxetic acid.

Dilution by doubling the volume, plus slow injection, prolonged the arterial-phase transit time and gave a more uniform shape to the bolus, allowing a more homogeneous intravascular contrast concentration during k-space data sampling [20, 30].

Furthermore, from Zech et al [24], who found higher SNR at 1 ml/s than at 2 ml/s in AP images of pigs injected with gadoxetic acid, we recognize the effect of slow injection. Likewise, Ringe et al [25] among others found that arterial-phase SNR in the aorta and portal vein were exceedingly higher in both slow injection groups, i.e., 1 ml/s (*p* < 0.0001 and *p* = 0.0016) [31]. However, neither author analyzed the SI values in the liver parenchyma.

There is a discrepancy between our results and some previous publications, which did not find significant differences in liver SNR between the two protocols, i.e., ND and D exams [15, 32]. However, we attribute our results to the combination of dilution and slow injection, as no previous studies combined both techniques. Furthermore, gadoxetic acid’s relatively high relaxivity in plasma likely explains why SI and SI_Norm_ remained so high, despite the further dilution of its already relatively small per kilogram dose, i.e., 0.025 mmol/kg versus 0.1 mmol/kg dose of gadolinium chelates [23] [33] [32]. Stretching the bolus, by dilution and slow injection, allows more time for protein binding of gadoxetic acid to human plasma. Since the bound fraction of gadoxetic acid has higher relaxivity than the unbound portion, the less compact bolus results in increased relaxivity [32, 33].

Additionally, increasing SI_Norm_ in the HBP is partly due to recirculation of intravascular gadoxetic acid that has not yet gone to the kidney for clearance [34], as the half-life is about 1 h [27–29]. We emphasize that during recirculation, the D exams continue to maintain their advantage over the ND exams, i.e., relatively higher SI_Norm_. We assume that after the intravascular phase, hepatocyte transporters had more time to accumulate gadoxetic acid due to doubling the volume on the diluted exams.

In general, OATP is a low-affinity but high-capacity carrier for gadoxetic acid [35]. This favors rapid liver enhancement [36]. Because of the bidirectional activity of the OATP transporter, until its decay, any gadoxetic acid not used by the hepatocyte re-enters the intravascular space, giving the remaining gadoxetic acid repeated chances to be taken up by OATP during recirculation [35]. Thus, OATP transporters on the diluted exams had twice as long as the non-diluted group to take up gadoxetic acid, due to the doubled volume caused by saline dilution, and the fact that there is no saturation effect for gadoxetic acid at clinically injected doses [37–40]. Thus, SI_Norm_ could continuously increase, being significantly higher on the D versus ND exams in almost all phases.

Therefore, dilution and slow injection plus shorter scan times seem a very good strategy to mitigate AP artifacts and preserve high-quality imaging [7, 14, 16, 41]. To the best of our knowledge, our study is the first dedicated to elucidating this important issue.

Our study had several limitations. First, it was a retrospective study, with a relatively small cohort and especially few comparable lesions, as the majority of the patients with HCC and metastases underwent treatment leading to a change in the appearance of these lesions between ND and D exams. However, it was sufficient to attain results of statistical significance. Secondly, despite the 5-year interval, we kept all imaging parameters almost constant for both groups.

Second, the fact that we found no significant difference in SI and SI_Norm_ on initial and follow-up unenhanced scans, i.e., our reference images, supports our conclusion that the increase in all phases except transitional was due to dilution and slow injection. Furthermore, even if some parameters, such as FOV, matrix size, and slice thickness, were not constant for the entire cohort as they depend on patient size, they were almost identical for each patient (D vs ND). Therefore, there was no bias since each patient was being compared to himself.

Third, although we initially calculated SNR and CNR, we found extreme outliers since the SD values for air were highly variable throughout the FOV. Furthermore, Dietrich et al [42] have criticized SNR since its calculation for any two ROI on a single image will, in general, not correlate with the true SNR measured on the image after applying certain reconstruction filters, multi-channel reconstruction, or parallel imaging. On the contrary, by using *C*_Norm_, we achieved very stable and consistent values that seemed credible, i.e., representative of the actual lesion visibility. The *C*_Norm,_ like CNR, is an absolute value. It describes the magnitude of the difference between a lesion and the adjacent liver parenchyma without providing information about whether the lesion enhances more or less than the surrounding liver parenchyma after gadoxetic acid injection. In other words, *C*_Norms_ intuitively provide information about lesion visibility, regardless of whether a focal lesion is hypointense or hyperintense. This enabled an evaluation of the effect of dilution and slow injection on focal liver lesion visibility.

Fourth, the diluted group of patients and technicians were already familiar with the sensation post-gadoxetic acid injection, a potential advantage and bias favoring the diluted vs. non-diluted patients [43]. However, this is a controversial point. Based upon the literature, Wybranski et al [44] reported that TSM artifacts cannot be mitigated by education and training.

Furthermore, several researchers cite that a previous episode of TSM is a risk factor for another episode of TSM on subsequent gadoxetic acid administration [3, 6, 44, 45].

Next, we used a test bolus injection which is not ideal as it may affect subsequent signal intensity following the intended injection. As we did not have the automatic bolus software, we instead used a 5-s delay for both D and ND groups to estimate peak enhancement, followed by identical scanning parameters for both groups. Choosing the same delay when doubling the injection time was not the optimal protocol. Since we compared each patient to himself for measuring SI_Norm_ differences on D versus ND exams, there was no inherent bias with the test bolus. Even though we may have performed the D group exams too early, the SI values were still higher for D vs ND. It could be speculated that if we had started imaging at t-peak plus 10 s, the SI would probably have been even higher for D.

Lastly, we did not analyze the specific clinical conditions that predisposed patients to AP artifacts. By again comparing each patient to himself, we eliminated the influence of gender, weight, and BMI that can contribute to severe AP artifacts. Nevertheless, due to the retrospective nature of our study, a prospective study, ideally in a larger cohort, is warranted to confirm our findings, especially regarding focal liver lesions.

In conclusion, our results suggest that 1:1 saline dilution of gadoxetic acid, administered at a slowed injection rate of 1 ml/s via a power injector, reduces AP artifacts while increasing SI_Norm_ and preserving *C*_Norm_ of images.

## Abbreviations

Abs: Absolute
AP: Arterial phase
BMI: Body mass index
*C*_Norm_: Contrast-to-norm ratio
D: Diluted imaging protocol
DTPA: Diethylenetriamine penta-acetic acid
FOV: Field of view
HASTE: Half-Fourier single-shot turbo spin-echo
HBP: Hepatobiliary phase
ICC: Intraclass correlation coefficient
IV: Intravenous
MRI: Magnetic resonance imaging
ND: Non-diluted imaging protocol
PACS: Picture archiving and communication system
ROI: Region of interest
SD: Standard deviation
SI: Signal intensity
SI_Norm_: Signal-to-norm ratio
STROBE: Strengthening the Reporting of Observational Studies in Epidemiology
T: Tesla
TSM: Transient severe motion artifact
VIBE: Volumetric Interpolated Breath-hold Examination

## Declarations

### Guarantor

The scientific guarantor of this publication is Univ.-Prof. Univ.-Doz. Dr. Ahmed Ba-Ssalamah.

### Statistics and Biometry

Dr. Michael Weber is a professional statistician. He has performed the statistics for this study. He is one of the authors.

michael.weber@meduniwien.ac.at

### Study subjects or cohorts overlap

Thirty of these subjects have been previously used for the following article.

J Magn Resonance Imaging 46 (4), 1107-1114 Oct 2017

Respiratory Motion Artifacts During Arterial Phase Imaging with Gadoxetic Acid: Can the Injection Protocol Minimize This Drawback?

Stephan H Polanec, Hubert Bickel, Pascal A T Baltzer, Patrick Thurner, Florian Gittler, Jacqueline C Hodge, Mustafa R Bashir, Ahmed Ba-Ssalamah

### Methodology

- retrospective
- observational
- performed at one institution
